# The impact of strike action on patient morbidity: A systematic literature review

**DOI:** 10.1002/hpm.3418

**Published:** 2022-01-18

**Authors:** Ryan Essex, Wendy Milligan, Gareth Williams, Sharon Marie Weldon

**Affiliations:** ^1^ The Institute for Lifecourse Development University of Greenwich London UK; ^2^ School of Health Sciences University of Greenwich London UK

**Keywords:** morbidity, protest, strike

## Abstract

Strike action in healthcare has been common over the last several decades. The overarching aim of this systematic review was to synthesise and analyse the empirical literature that examines the impact of strike action on patient morbidity, that is, all patient outcomes except mortality. After conducting a search and apply eligibility criteria, 15 studies were included in this review. These articles included a variety of outcomes from hypertension control to rates of chlamydia. Strikes ranged from 13 to 118 days, with a mean strike length of 56 days. A textual narrative synthesis was employed to arrange studies by whether they had a positive, mixed or neutral or negative impact on patient morbidity. Results suggest that strike action has little impact on patient morbidity. The majority of studies reported that strike action had a neutral or mixed impact of strike action on patient morbidity. One study reported positive outcomes and three studies reported negative outcomes, however in both cases, the impact that the strike had was marginal.

## Highlights

- Strike action is widely debated with a major concern related to patient outcomes, including patient morbidity
- Strike action has little impact on patient morbidity, almost all studies reported no adverse outcomes
- Strike action by healthcare staff can be carried out safely in relation to patient morbidity

## 1 INTRODUCTION

Strike action in healthcare has been common over the last several decades. Strikes have occurred on almost every continent, for a range of reasons. They have been carried out over a matter of hours to hundreds of days. While healthcare strikes raise a range of issues, one of the most pressing that is almost always raised relates to the impact that the action could have on patient wellbeing. That is, most debates centre on the impact that strike action could have on patients, with those arguing both for and against such action citing patient safety as a major concern.^1^ These concerns have some basis as strikes, by definition are designed to disrupt the delivery of care.

Over the years there has been a growing body of evidence that has examined the impact of strike action on the health and wellbeing of patients. The majority of this evidence has examined patient mortality, with evidence suggesting that generally, strikes do not significantly change patient mortality in‐hospital^2^ or more generally when looking at population based statistics, for example.^3^ While mortality is an important variable to consider, focussing on it alone overlooks a number of other patient outcomes that may be impacted by strike action; because of this, this review will focus only the impact of strike action patient morbidity.

The overarching aim of this review is to synthesise and analyse the empirical literature that examines the impact of strike action on patient morbidity, that is, all patient outcomes except mortality. This reviews seeks to (1) understand if strike action has an impact on morbidity and if so (2) what factors related to the strike, or the health of patients in particular impact these outcomes.

## 2 METHODS

### 2.1 Design

A systematic review was employed to identify and synthesise all relevant literature in relation to the above research questions. PRISMA and ENTREQ reporting guidelines were followed.^4^, ^5^ This review follows a results‐based convergent synthesis design meaning that qualitative, quantitative and mixed‐methods studies are identified in a single search, presented, reported and analysed separately, and integrated during data summary and synthesis.^6^, ^7^ In conducting this review the following steps were followed: (1) systematic literature search, (2) data extraction, (3) quality appraisal, (4) data synthesis and presentation. These steps are outlined below.

### 2.2 Search strategy

The following electronic databases and time periods were searched: EMBASE (1980–2021), MEDLINE (1946–2021), CINAHL (1982–2001), BIOETHICSLINE (1972–1999), EconLit (1969–2021), WEB OF SCIENCE (1960–2021). In addition, grey literature was searched through OPEN GREY, and SIGMA REPOSITORY. Search terms were developed to capture the core concepts related to the form of intervention we were interested in (e.g., strike action, industrial action) and the populations in question (e.g., doctors, nurses, health workers). The final search terms were: strike OR ‘industrial action’ OR ‘industrial dispute’ OR ‘collective action’ AND doctor OR physician OR clinician OR ‘medical practitioner’ OR nurs* OR ‘health profession*’ OR healthcare OR ‘health care’ OR ‘pharmac*’ OR ‘dentist’ OR ‘midwi*’ OR dieti* OR ‘occupational therap*’ OR ‘paramed*’ OR ‘physiotherap*’ OR ‘radiograph*’ OR ‘psycholog*’ OR ‘health worker’ OR ‘hospital’. There were no publication dates or language restrictions. Where complete data for a relevant outcomes was not available we contacted authors to request data. In addition, we conducted a manual search of the reference lists of eligible studies.

### 2.3 Search inclusion/exclusion criteria

**FIGURE 1:**
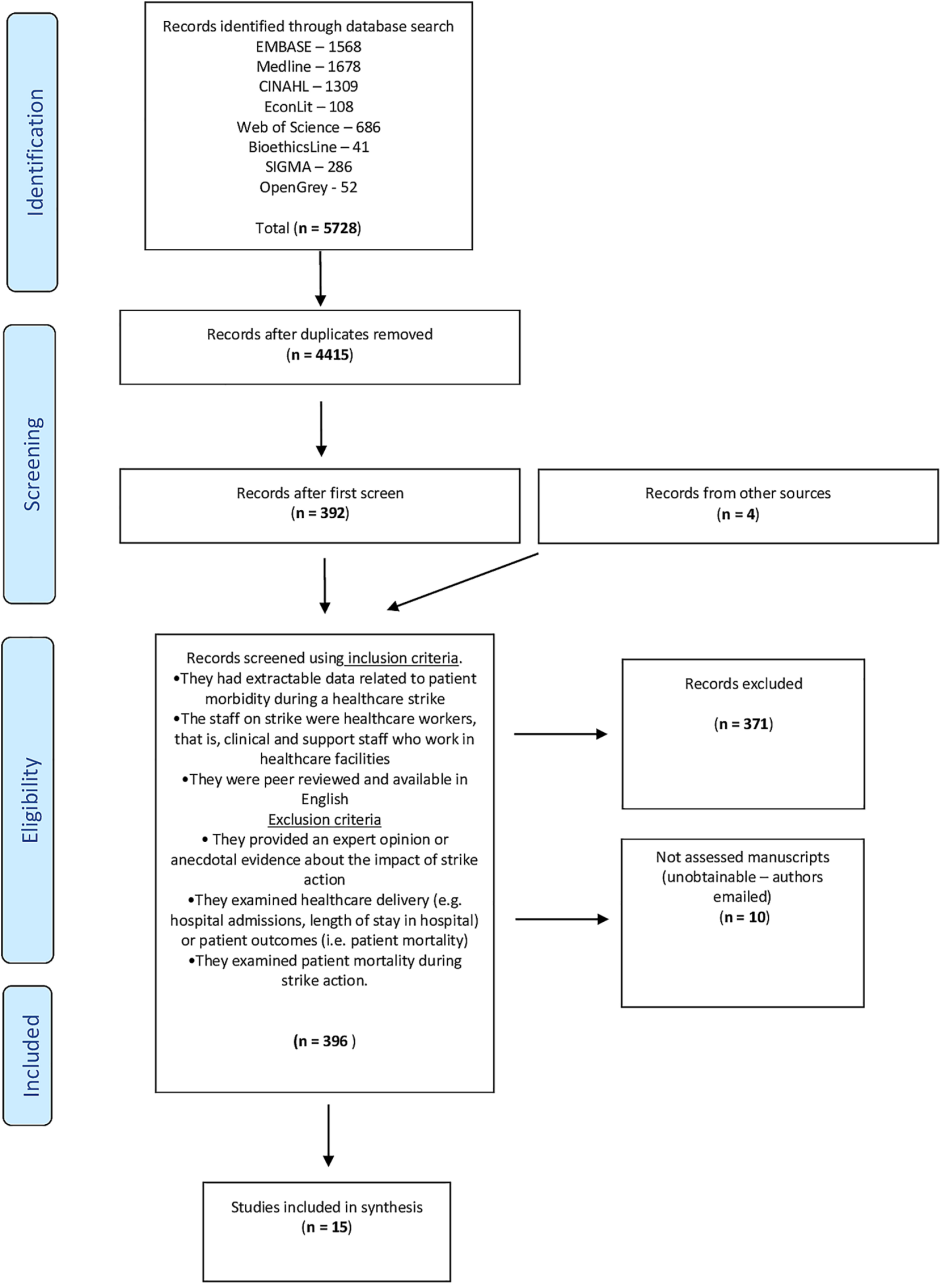
PRISMA flow diagram

The initial search returned 5728 results, which were imported into Endnote where duplicates were removed. This left 4415 articles. The title and abstract of these articles was scanned and articles not meeting the inclusion criteria were removed. After the initial screen, 392 articles remained and a second full‐text screen was undertaken and reference lists were searched. A further four papers were found and all 396 articles were assessed against the below eligibility criteria, leaving 15 articles (see Figure 1).

Papers were included if:

- The staff on strike were healthcare workers, that is, clinical and support staff who work in healthcare facilities.
- They included empirical data.
- They had extractable data related to patient morbidity during a healthcare strike.
- They were peer reviewed.

Papers were excluded if:

- They provided an expert opinion or anecdotal evidence about the impact of strike action.
- They were literature reviews of any sort.
- They examined healthcare delivery (e.g., hospital admissions, length of stay in hospital) or patient mortality.
- They examined patient mortality during strike action.

### 2.4 Data extraction

Data from the included studies was extracted by RE, checked by SMW and categorised according to the source, country of where the research took place, study aims and objectives, research methods/design, the context of the study, nature of the strike, main outcomes, and quality appraisal scores and issues (see Table 1). Categories were kept broad due to methodological differences across and within studies and therefore summary measures were not possible.

**TABLE 1:** Articles included in this review

Author	Year	Country	Aims of study	Methods	Study setting	Nature of strike	Staff on strike	Length of strike	Outcomes measured	Outcomes of study	Impact of strike	QA score
Bhattacharyya et al.	1980	UK	This study examined the impact of an ambulance staff strike on elderly day hospital patients	Quantitative—cross sectional survey	This study was conducted in a day centre in Northampton, with an average daily attendance of 42.	This action occurred in 1979 when industrial action by ambulance staff curtailed attendance at day hospitals.	Ambulance staff	63 days	Mobility and self‐care & mental state (along with other factors related to attendance and social support)	This study reported marginal impact on patients mental state, however this was a small sample and no tests for significance were conducted.	Neutral	n/a
Crocker et al.	2007	Canada	This study examined whether the prevalence of pneumonia changed during a doctor strike.	Quantitative—retrospective observational study.	This study was conducted in the Janeway Children's health and Rehabilitation Centre	This action occurred for 16 days, 1 October to 17 October 2002 when doctors in Newfoundland and Labrador went on strike.	Doctors	16 days	Pneumonia	This study reported no significant changes in diagnoses of pneumonia during the strike.	Neutral	2/9
Daga and Shende	1999	India	This study examined the impact of a strike on mortality and neonatal care during a junior doctors strike.	Quantitative—retrospective observational study.	Not stated in manuscript	This action began on 8 November 1991 and lasted 69 days. Several contingencies were put in place to maintain the clinic during the strike.	Doctors	69 days	High risk deliveries	This study reported no significant difference in the number of high‐risk deliveries during the strike.	Neutral	5/9
Dierssen et al.	1997	Spain	This study examined the risk of nosocomial infection during a strike in a hospital surgical department	Quantitative—retrospective observational study.	The study was done at the Sierrallana hospital, a 200‐bed secondary centre, with a referral population of 150,000 people.	This action was national and took place in 1995 from 8 May to 26 June.	Doctors	50 days	Nosocomial infection	This study reported no significant changes in the risk of all nosocomial infections during the strike period.	Neutral	3/9
Marcovici et. al.	1987	Israel	This study examined the effect of the strike on blood pressure control in a defined population of known ambulatory hypertensive patients.	Quantitative—retrospective observational study.	This study was carried out in Ashdod, where 73% of patients were insured by Kupat Holim. During the strike Kupat Holim clinics were open and staffed by nurses, who carried out their usual follow‐up activities.	This action occurred in 1983 over 118 days. All doctors in government facilities went on strike. The Israel Medical Association established ad hoc health centres all over the country to provide medical care on a fee‐for‐service basis. This survey was conducted in the last 2 weeks of the strike	Doctors	118 days	Hypertension control	This study reported that the strike was associated with a measurable increase in the prevalence of uncontrolled hypertension which was limited to males from higher socioeconomic backgrounds.	Mixed	3/9
Njuguna	2018	Kenya	This study examined the impact of a nurse strike on immunization services.	Quantitative—retrospective observational study.	This study used population based data (from all of Kenya) on vaccination rates.	This action occurred amongst broader unrest prior to the strike by nurses, doctors had been on strike for 100 days. Nurses went on strike after doctors had returned to work on June 5, 2017. The strike lasted for 5 months.	Nurses	150 days	Immunisation rates	This study reported a significant decline in vaccinated infants during the strike period, with a 56.9% decline.	Negative	5/9
Norman and Malla	1984	Canada	This study examined the impact of a strike on an in‐patient psychiatric hospital.	Quantitative—retrospective observational study.	This study occurred in St Johns hospital, Newfoundland. The psychiatric ward had almost 400 beds including 55 acute beds.	This strike occurred in Feb 1977 and lasted 17 weeks, only a few admissions were made during this time.	“Non‐professional employees”	119 days	Mental health—violent and suicidal behaviour	This study reported that there were a significant decrease in admissions, but there was an increase in the proportion of involuntary admissions or those showing suicidal or violent behaviour.	Mixed	6/9
Prinsley	1971	UK	This study examined the impact of an ambulance service strike on day hospital patients	Quantitative—retrospective observational study.	This study was carried out in a day hospital in Teeside.	This strike occurred in Oct 1970 when ambulance staff went on strike. For five weeks they only carried emergencies. Patients who attended this day hospital were taken by taxi or relatives.	Ambulance staff	35 days	Deterioration of various conditions (i.e., diabetes, arthritis, cerebral thrombosis)	This study reported that almost 10% of patients failed to return for treatment after the strike concluded. The authors concluded that there were a number of extra admissions to the wards because of the strike.	Negative	3/9
Sigal et. al.	1989	Israel	This study examined the impact of a nursing strike on hospitalised patients with schizophrenia.	Quantitative—retrospective observational study.	This study was carried out in Pardessia Government Hospital.	This action occurred on Jun 23 1986 when a nationwide nurses’ strike occurred in Israel. No nurses were on duty during that period. Other members of staff assumed some of the nurses’ responsibilities.	Nurses	17 days	Mental health—functioning of patients on psychiatric ward	This study reported that patients showed more responsibility and greaten participation in ward maintenance, increased initiative, and functioned more independently during the strike.	Positive	3/9
Stovall et. al.	2004	USA	This study examined the impact of a strike on a community mental health centre.	Case report from clinicians who worked within the institution.	This study was carried out in a community mental health centre in Worcester, Massachusetts.	This action occurred in Oct 1999. All employees who were represented by the union and who worked in the detoxification units, residential programs, and urban adult and children’s clinics went on strike. Staff who did not strike took on additional duties.	Healthcare workers (Nurses, social workers, case workers and residential staff)	30 days	Mental and general health of patients	This study reported that while the strike had a significant impact on staff and the administrative side of the centre, patients were not harmed by the strike.	Neutral	n/a
Mustard et al.	1995	Canada	This study examined the impact of a 31‐day nurses' strike on the cesarean birth rate in the province of Manitoba, Canada.	Quantitative—retrospective observational study.	This study explored all Manitoba based hospitals, 57 of 87 provided obstetric services.	This action occurred on Jan 1, 1991 when nurses withdrew nonessential services for 31 days, During the strike major obstetric facilities continued to provide care with approximately 30% of their normal nursing staff complement.	Nurses	31 days	Caesarean birth rates/adverse newborn outcomes	This study reported a decreased rate of caesarean sections during the strike, however this study also detected an increase in the pooled incidence of adverse newborn outcomes. presentation and among women with previous caesarean section.	Mixed	6/9
Pinto et al.	2013	Canada	This study examined the impact of a strike on sexual health services.	Quantitative—retrospective observational study.	This study used population data to examine the incidence of reported chlamydia in Toronto during strike and non‐strike periods.	This action occurred on Jun 22 2009 when, staff from the Toronto municipal sexual health programme went on strike. Five of the 95 employees continued to work during the strike.	Toronto municipal workers for a sexual health programme	36 days	Chlamydia	This study reported no significant difference in chlamydia incidence, except among females under 25 years old immediately following the strike.	Mixed	6/9
Pantell and Irwin	1979	USA	This study examined the impact that a strike had on appendectomies	Quantitative—retrospective observational study.	This study examined data from 13 hospitals in San Francisco county who carried out appendectomies.	This action occurred on 1 May 1975 when doctors went on strike. The authors reported this caused major changes in patterns of providing surgical care; only emergency surgery was provided.	Doctors	30 days	Inflamed appendices/appendectomies	This study reported there were no changes in appendectomies performed during the strike. Furthermore, the ratio of normal to inflamed appendices was no different.	Neutral	7/9
Aro and Hosia	1987	Finland	This study examined the impact of a strike on the utilisation of services, but also on a range of diagnoses and indicators of patient wellbeing	Quantitative—retrospective observational study.	This study occurred in Varkaus Health Centre which serves the city of Varkaus and two smaller rural communities, at the time of the study the total population was 33,000.	This action occurred on 5 Apr 1984, when doctors went on strike for seven weeks. This study only reports on the latter stages of the strike, when doctors from the Varkaus health centre joined. Normally, the centre employed 16 doctors, during the strike 3 doctors were on call for urgent matters.	Doctors	13 days	Various diagnoses. Respiratory, otitis media, abdominal , diabetes, low back pain, etc.	This study reported that the population had little difficulty in adapting to the short‐term reduction of services with no evidence of harmful effects of the strike.	Neutral	4/9
Kofoed et al.	2009	Denmark	This study examined the impact of a nurse strike on paediatric diabetes control.	Quantitative—retrospective observational study.	This study occurred in Kolding hospital. Little further information is provided.	Not stated—however the strike occurred in 2008.	Nurse	60 days	Diabetic control	This study reported higher HbA1c values post‐strike, suggesting that the strike resulted in poorer diabetic control amongst a number of children.	Negative	4/9

### 2.5 Quality appraisal

Studies were appraised by WM and GW utilising the Newcastle–Ottawa Scale (NOS).^8^ This scale was developed to examine the quality of case control and cohort studies, with studies judged on three areas: study population (and cases or controls), the comparability of these groups, and the outcome of interest or ascertainment of exposure. Studies are scored out of nine with higher scores indicating generally higher quality studies.

### 2.6 Data summary and synthesis

Studies were combined to summarise descriptive statistics of the study characteristics, followed by a textual narrative synthesis. This approach arranges disparate study types into more homogenous sub‐groups which aids in the synthesising of different types of evidence and in this case, answering research questions which can be informed by multiple methodological approaches.^9^

## 3 RESULTS

The 15 articles included measured a variety of outcomes from hypertension control to rates of chlamydia. The articles also included substantial geographic diversity with studies from Europe, North America, Africa and Asia. Six studies examined strikes by doctors, four examined strikes by nurses, with the remainder of studies examining strikes by ambulance staff, ‘non‐professional employees’, government employees and multiple healthcare staff from a mental health centre. Strikes ranged from 13 to 118 days, with a mean strike length of 56 days. These results are summarised in Table 1.

### 3.1 Quality appraisal

Thirteen of the papers included in this review were reviewed against the criteria set out in the NOS. Two papers were excluded as they did not utilise observational designs.^10^, ^11^ The quality of the 13 remaining papers varied. Four studies scored well, scoring six or above.^12^, ^13^, ^14^, ^15^ Several studies scored relatively low on this scale, with five papers scoring three or less.^16^, ^17^, ^18^, ^19^, ^20^ The remainder of these studies fell somewhere between, scoring four or five.^21^, ^22^, ^23^, ^24^ Collectively, the majority of papers lacked detail relating to the representativeness of the exposed cohort (and to a lesser degree the selection of the non‐exposed cohort), the comparability of the cohorts and the follow up related to the cohorts. As a whole, these results suggest that amongst the studies included in this review only a few could be considered high‐quality and therefore at minimal risk of bias.

### 3.2 The impact of strike action on morbidity

Papers were categorised into whether they had a negative, mixed or neutral or positive impact on patient morbidity. Studies were categorised as having a negative impact when they reported worse patient outcomes during a strike. Studies were categorised as neutral when they reported no substantial impact on patient outcomes during a strike. And studies were categorised as positive when they reported an improvement in patient outcomes during a strike. Studies were categorised as mixed when they reported a mixture of positive, negative or neutral results. There were three studies that reported a clear negative impact. These studies varied substantially. There were four studies that reported a mixed impact of strike action. Amongst these studies, three were conducted in Canada and one in Israel. One Israeli study reported a positive impact of strike action. There were no obvious patterns that linked these outcomes to the nature of the strike, for example, the length of strike, when or where the strike occurred.

### 3.3 Studies reporting a negative impact of strike action

There were three studies that reported a clear negative impact. These studies varied substantially. These were carried out in Kenya, Denmark and the UK. One involved ambulance staff, the two involved nursing staff. The strike in Kenya lasted 100 days, the strike in Denmark lasted 60 days, while the strike in the UK last 35 days. The studies examined the impact on immunisation services, diabetic control and more general outcomes from a day hospital.

Taking a closer look at the first of these studies, Njuguna^24^ examined the impact of a nurse strike on immunisation services. This strike occurred amongst broader unrest. Prior to the strike by nurses, doctors had been on strike for 100 days. Nurses went on strike after doctors had returned to work on 5 June 2017 and the strike lasted for 5 months. This study reported a significant decline in vaccinated infants during the strike period, with a 56.9% decline. The study also noted that during the same period of time, faith‐based health services (which were not on strike) reported a 251.6% increase of immunisation rates during the strike period. This study provides no details on whether vaccine‐preventable disease increased as a result of the strike. While this study shows a clear negative impact, the study carried out in the UK by Prinsley^18^ is somewhat more difficult to interpret. This study was carried out in the context of a 35 day ambulance strike in a day hospital. During the time the day hospital functioned as normal, however patients could not attend unless taken by taxi or relatives. While this study ran no significance tests, the authors concluded that there were a number of extra admissions to the wards because of a lack of diagnostic and treatment facilities in the day hospital. Furthermore, this study reported that almost 10% of patients failed to return for treatment after the strike concluded. Finally, Kofoed et al.^22^ examined the impact of a nurse strike on paediatric diabetes control during a 60 day strike that occurred in Denmark in 2008. This study found higher HbA1c values post‐strike, suggesting that the strike resulted in poorer diabetic control amongst a number of children.

### 3.4 Studies reporting a neutral or mixed impact of strike action

There were four studies that reported a mixed impact of strike action. Amongst these studies, three were conducted in Canada and one in Israel. Strikes ranged in length from 31 to 118 days. Each study examined a different group of workers; doctors, nurses, ‘non‐professional’ health workers and sexual health programme workers. The outcomes examined include hypertension control, mental health, caesarean birth rates and prevalence of Chlamydia.

In one of the earliest studies Norman and Malla^12^ examined the impact of a strike on an in‐patient psychiatric hospital. This strike occurred in February 1977, involved ‘non professional’ workers and lasted 17 weeks. According to this study only a few admissions were made during this time. While this study reported a significant decreased in admissions overall, there was an increase involuntary admissions and patients exhibiting violent behaviour. However, when looking at general hospital admissions (where patients were diverted) admission patterns, along with the number of patients exhibiting violent behaviour were largely similar. A study by Marcovici et al. conducted in the 1980s examined the impact of a doctors strike on hypertension control.^17^ This strike in Israel occurred over 118 days and during this period clinics were staffed by nurses. This study was carried out in the last 2 weeks of the strike with results suggesting no changes in hypertension control for women or men from lower socioeconomic backgrounds. An increase in uncontrolled hypertension was only observed in men from higher socioeconomic backgrounds. In a further that reported mixed results, Mustard et al.^13^ examined the impact of a 31 day nurses' strike on the caesarean birth rate in Canada. This study explored all Manitoba based hospitals; 57 of 87 provided obstetric services. This strike occurred on 1 Jan 1991 when 10,500 members of the Manitoba Nurses Union withdrew nonessential services for 31 days. During the strike the major obstetric facilities in the province continued to provide care with approximately 30% of their normal nursing staff complement. This study reported a decreased rate of caesarean sections during the strike, however this study also detected an increase in the pooled incidence of adverse new born outcomes. Furthermore, in response to constraints imposed by a reduced nursing complement, doctors increased the frequency of vaginal birth in breech presentation and among women with previous caesarean section. One final and more recent study also reported more mixed results, exploring the impact of a strike on sexual health services.^14^ This study used population data to examine the incidence of reported chlamydia in Toronto during strike and non‐strike periods. This strike lasted 36 days, with 5 of the 95 staff continuing to work to provide care for those with more acute needs. This study reported that overall there was no significant difference in chlamydia incidence during the strike, however there was a small but significant increase in the incidence of chlamydia amongst females under 25 years old immediately following the strike.

There were seven studies that reported a neutral impact of strike action. These studies were again diverse conducted in the UK, Canada, India, Spain, Finland and two in the US. Strikes varied in length from 13 days in Finland to 69 days in India. Five studies examined strikes that involved doctors; two studies examined strikes that involved ambulance workers and healthcare staff. The outcomes examined included paediatric pneumonia, high‐risk deliveries, nosocomial infection, appendectomies. Three studies examined more general mental and general health outcomes or multiple outcomes.

Looking more closely at these studies, Crocker et al.^20^ reported no significant changes in diagnoses of paediatric pneumonia during a 16 day doctor strike in Canada. Daga and Shende^23^ reported no significant difference in the number of high‐risk deliveries during a 69 day junior doctor strike in India. Dierssen et al.^16^ reported no significant changes in the risk of all nosocomial infections (and surgical site infections) during a 50 day doctor strike in Spain. Similarly, Pantell and Irwin^15^ reported there were no changes in appendectomies performed, inflamed appendices or delays in surgery during a 30 day doctor strike in the US. Three further studies examined more general or multiple outcomes. In a cross‐sectional survey Bhattacharyya et al.^11^ asked patients in a day hospital in the UK to self‐rate their health during an ambulance staff strike which lasted 63 days. This study reported marginal impact on patients mental state, however this was a small sample and no tests for significance were conducted. Stovall et al.^10^ similarly concluded, that during a 30 day ‘healthcare worker’ strike, while the strike had a significant impact on staff and the administrative side of the centre, patients were not harmed by the strike. Finally, Aro and Hosia^21^ examined the impact of a 60 day nursing strike on the utilisation of services, but also on a range of diagnoses and indicators of patient wellbeing. This study reported that the population had little difficulty in adapting to substantial short‐term reduction of ambulatory services with no evidence of harmful effects of the strike.

### 3.5 Studies reporting a positive impact of strike action

One study reported a positive impact of strike action. Sigal et al.^19^ examined the impact of a nursing strike on hospitalised patients with schizophrenia in a government hospital during an Israeli nursing strike. In this hospital, no nurses were on duty. Other members of the staff, including a psychiatrist, a clinical psychologist, and a social worker, assumed some of the nurses' responsibilities. The authors report that from early afternoon until morning, the 29 chronic schizophrenic patients on the unit were practically on their own for the 17 days of the strike. A questionnaire was developed that included questions related to patient responsibilities, initiative and helpfulness. Patients were observed by staff during the strike and non‐strike period. This study reported that patients showed more responsibility towards ward property and other patients and greater participation in ward maintenance during the strike. They also showed increased initiative, offered help more frequently, and functioned more independently.

## 4 DISCUSSION

This paper sought to synthesise and analyse the empirical literature on the impact of strike action on patient morbidity in an effort to understand if strike action has an impact on morbidity and if so what factors related to the strike or patients impacted these outcomes. As a whole, the literature suggests that strike action has little impact on patient morbidity. The majority of studies reported that strike action had a neutral or mixed impact of strike action on patient morbidity. One study reported positive outcomes and three studies reported negative outcomes, in each case however and with the exception of Njuguna^24^ who examined immunisation rates, the negative impacts reported were marginal. Few patterns emerged that seemed related to patient outcomes. That is, the nature of the strike, the country in which it took place, the professions on strike didn't seem to impact on whether a strike had a negative, neutral or positive impact on patient morbidity. Across the studies that reported negative, neutral or positive results, all varied substantially. Furthermore, a substantial number of studies included in this review had significant issues related to quality and were at risk of bias, for this reason, these results should also be treated with caution. In saying this however, given these results, it seems relatively safe to conclude that in regard to morbidity, strikes can be conducted safely, the factors that ensure this is the case are less clear.

The studies included in this review were relatively heterogeneous in the outcomes they examined and the context in which they occurred, so there is a general need for caution in how these results are interpreted. Furthermore, we have been deliberately broad in regards to the studies included here. Some overlap substantially with the provision of services. For example, it is arguable that some studies measure disruption to service rather than patient outcomes. For example, while Njuguna^24^ reported a decreased number of vaccinations because of a strike, which is undoubtedly a negative result, it could be argued this is more a service disruption; this study provides no details on whether vaccine‐preventable disease increased as a result of the strike. Caution is warranted elsewhere. Closely related to this point is the question of how directly strike action impacted on patient outcomes. That is, while some impacts were more clear cut, like Njuguna^24^ above, this cannot be said of all studies. For example, while Norman and Malla^12^ reported an increase in the rate of violent and involuntary psychiatric admissions during a strike (i.e., as a proportion of overall admissions), this appeared to have little to do with the strike itself; it may have been that voluntary or less acute patients put off seeking treatment which resulted in more acute patients making up a greater portion of overall patients during the strike. Finally, in saying this, we cannot completely rule out strike action having an impact on patient outcomes. While some studies did a relatively good job at reporting the context and nature of strike action, many others did not, making it difficult to gain further insight into the impact of the different features of strike action on patient outcomes.

Over the last few years and since the COVID‐19 was declared a global pandemic strike action appears to have become increasingly common across the globe.^25^ Debates about the justifiability of strike action are likely to become increasingly pressing, particularly while the world comes to terms with the long‐term impacts of COVID‐19. Fortunately, and unlike many other forms of adversarial action in healthcare, we can measure the impact of strike action. While strikes should be planned carefully and while careful consideration should be given to patient wellbeing, this review provides similar evidence to what is often found in relation to mortality, that is, that strikes have a negligible impact on patient wellbeing. Further research is needed however to examine a broader range of patient outcomes and to better understand how patients utilise services during strike action.

## CONFLICT OF INTEREST

The authors declare that they have no competing interests.

## ETHICS STATEMENT

Ethics approval was not sought or required for this study.

## Data Availability

There is no dataset associated with this study.

## References

[1] Essex R , Weldon S . Strike action in healthcare: a systematic critical interpretive synthesis. Nurs Ethics. In press. 10.1177/09697330211022411 PMC9442631 35411830

[2] Cunningham SA , Mitchell K , Narayan KM , Yusuf S . Doctors' strikes and mortality: a review. Soc Sci Med (1982). 2008;67(11):1784‐1788. (Epub 2008 Oct 2008). 10.1016/j.socscimed.2008.09.044 18849101

[3] Erceg M , Kujundzic‐Tiljak M , Babic‐Erceg A , Coric T , Lang S . Physicians' strike and general mortality: Croatia's experience of 2003. Coll Antropol. 2007;31(3):891‐895. 18041403

[4] Page M , McKenzie J , Bossuyt P , Boutron I , Hoffmann T . The PRISMA 2020 statement: an updated guideline for reporting systematic reviews. Br Med J. 2021. 10.1371/journal.pmed.1003583 PMC8007028 33780438

[5] Tong A , Flemming K , McInnes E , Oliver S , Craig J . Enhancing transparency in reporting the synthesis of qualitative research: ENTREQ. BMC Med Res Methodol. 2012;12(1):181. 23185978 10.1186/1471-2288-12-181 PMC3552766

[6] Noyes J , Booth A , Moore G , Flemming K , Tunçalp Ö , Shakibazadeh E . Synthesising quantitative and qualitative evidence to inform guidelines on complex interventions: clarifying the purposes, designs and outlining some methods. BMJ Glob Health. 2019;4(Suppl 1):e000893. 10.1136/bmjgh-2018-000893 PMC6350750 30775016

[7] Hong QN , Pluye P , Bujold M , Wassef M . Convergent and sequential synthesis designs: implications for conducting and reporting systematic reviews of qualitative and quantitative evidence. Syst Rev. 2017;6:61. 28335799 10.1186/s13643-017-0454-2 PMC5364694

[8] Wells GA , Shea B , O’Connell D , et al. The Newcastle‐Ottawa Scale (NOS) for Assessing the Quality of Nonrandomised Studies in Meta‐Analyses.

[9] Lucas PJ , Baird J , Arai L , Law C , Roberts HM . Worked examples of alternative methods for the synthesis of qualitative and quantitative research in systematic reviews. BMC Med Res Methodol. 2007;7(1):4. 17224044 10.1186/1471-2288-7-4 PMC1783856

[10] Stovall JG , Hobart M , Geller JL . The impact of an employees' strike on a community mental health center. Psychiatr Serv (Washington, DC). 2004;55(2):188‐191. 10.1176/appi.ps.55.2.188 14762247

[11] Bhattacharyya BK , Isherwood J , Sutcliffe RL . Survey of elderly day hospital patients during a period of industrial action. Age Ageing. 1980;9(2):106‐111. 7395654 10.1093/ageing/9.2.106

[12] Norman RM , Malla AK . The effect of a mental hospital strike on general hospital psychiatric services. Psychol Med. 1984;14(4):913‐921. 6545421 10.1017/s0033291700019875

[13] Mustard CA , Harman CR , Hall PF , Derksen S . Impact of a nurses' strike on the cesarean birth rate. Am J Obstet Gynecol. 1995;172(2 Pt 1):631‐637. 7856697 10.1016/0002-9378(95)90584-7

[14] Pinto AD , Gournis E , Al‐Bargash D , Shahin R . Impact of a labour disruption affecting local public health on the incidence of Chlamydia infections in Toronto. PLoS One. 2013;8(11):e79375. 24312180 10.1371/journal.pone.0079375 PMC3843662

[15] Pantell RH , Irwin CE . Appendectomies during physicians' boycott: analysis of surgical care. JAMA. 1979;242(15):1627‐1630. 480579

[16] Dierssen T , Farinas‐Alvarez C , Llorca J , Antolin FM , Delgado‐Rodriguez M . Risk of nosocomial infection during a 50‐day surgeon strike. J Hosp Infect. 1997;36(3):241‐243. 9253706 10.1016/s0195-6701(97)90200-0

[17] Marcovici OA , Slater PE , Ellencweig AY . Effects of the Israel doctors' strike on hypertension control in Ashdod. Eur J Epidemiol. 1987;3(1):30‐34. 3582596 10.1007/BF00145069

[18] Prinsley DM . Effects of industrial action by the ambulance service on day hospital patients. Br Med J. 1971;3(5767):170‐171. 5557871 10.1136/bmj.3.5767.170 PMC1800247

[19] Sigal M , Diamont I , Bacalu A , Arad L , Levi M . The effect of a nurses' strike on the functioning of chronic patients. Hosp Commun Psychiatry. 1989;40(4):409‐411. 10.1176/ps.40.4.409 2714756

[20] Crocker K , Cramer B , Hutchinson JM . Antibiotic availability and the prevalence of pediatric pneumonia during a physicians' strike. Can J Infect Dis Med Microbiol. 2007;18(3):189‐192. 18923715 10.1155/2007/138792 PMC2533544

[21] Aro S , Hosia P . Effects of a doctors' strike on primary care utilization in Varkaus, Finland. Scand J Prim Health Care. 1987;5(4):245‐251. 3423495 10.3109/02813438709018103

[22] Kofoed PE , Thomsen J , Ammentorp J . The influence of a hospital strike on the metabolic control of children with diabetes. Pediatr Diabetes. 2009;10(S11).

[23] Daga SR , Shende SR . Neonatal care during a residents' strike. Trop Dr. 1999;29(2):73‐75. 10.1177/004947559902900204 10418294

[24] Njuguna J . Impact of nurses' strike in Kenya on number of fully immunized infants in 18 county referral hospitals. J Health Care Poor Underserved. 2018;29(4):1281‐1287. 30449746 10.1353/hpu.2018.0095

[25] Essex R , Weldon S . Health care worker strikes and the covid pandemic. N Engl J Med. 2021. 10.1056/NEJMp2103327 33826818

